# RAvariome: a genetic risk variants database for rheumatoid arthritis based on assessment of reproducibility between or within human populations

**DOI:** 10.1093/database/bat073

**Published:** 2013-10-23

**Authors:** Yoko Nagai, Tadashi Imanishi

**Affiliations:** ^1^Department of Molecular Life Science, Division of Basic Medical Science and Molecular Medicine, Tokai University School of Medicine, 143 Shimokasuya, Isehara, Kanagawa 259-1193, Japan and ^2^Data Management and Integration Team, Molecular Profiling Research Center for Drug Discovery, National Institute of Advanced Industrial Science and Technology, Koto-ku, Tokyo 135-0064, Japan

## Abstract

Rheumatoid arthritis (RA) is a common autoimmune inflammatory disease of the joints and is caused by both genetic and environmental factors. In the past six years, genome-wide association studies (GWASs) have identified many risk variants associated with RA. However, not all associations reported from GWASs are reproduced when tested in follow-up studies. To establish a reliable set of RA risk variants, we systematically classified common variants identified in GWASs by the degree of reproducibility among independent studies. We collected comprehensive genetic associations from 90 papers of GWASs and meta-analysis. The genetic variants were assessed according to the statistical significance and reproducibility between or within nine geographical populations. As a result, 82 and 19 single nucleotide polymorphisms (SNPs) were confirmed as intra- and inter-population-reproduced variants, respectively. Interestingly, majority of the intra-population-reproduced variants from European and East Asian populations were not common in two populations, but their nearby genes appeared to be the components of common pathways. Furthermore, a tool to predict the individual’s genetic risk of RA was developed to facilitate personalized medicine and preventive health care. For further clinical researches, the list of reliable genetic variants of RA and the genetic risk prediction tool are provided by open access database RAvariome.

Database URL: http://hinv.jp/hinv/rav/

## Introduction

Rheumatoid arthritis (RA; MIM180300) is a common autoimmune disease characterized by the chronic inflammation of the bones and joints. Several epidemiological studies reported that RA prevalence varies among different populations (1). In North America and Northern Europe, the estimated prevalence of RA is 0.5–1.1%, but in Southern Europe, a lower prevalence of 0.3–0.7% has been reported. In East Asia, RA prevalence in the urban areas of Japan and Taiwan is 1.04% and 0.93%, respectively, but that in mainland China ranges from 0.2% to 0.37% (2, 3). Twin studies on RA have led to a heritability estimate for RA of 65% in the Finnish study and 53% in the UK study, and genetic factors account for an estimated 60% of the disease risk (4).

Large numbers of association studies have been conducted, and numerous single nucleotide polymorphisms (SNPs), genes and chromosomal regions have been reported to be associated with RA. Several SNP-trait association databases provide information about RA-associated loci, including the NHGRI Catalog of Published Genome-Wide Association Studies (GWASs) (5), GWASdb (6), GWAS Central (7), HuGE Navigator (8) and PharmGKB (9). On the other hand, some databases have been developed to provide detailed genetic information of a specific disease, e.g. AlzGene (http://www.alzgene.org/) for Alzheimer’s disease, CADgene (http://www.bioguo.org/CADgene/) for coronary artery disease and GOLD.db (https://gold.tugraz.at/) for lipid-associated disorders. There are a few databases focusing on autoimmune diseases; type 1 diabetes database T1Dbase (http://www.t1dbase.org) and Lymphoproliferative syndrome database ALPSbase (http://research.nhgri.nih.gov/ALPS/).

As the first human genetic variation database for RA, RAvariome aims to provide a reliable set of RA risk variants that was systematically assessed according to its reproducibility. Candidate gene association studies and GWASs are known to be vulnerable to a range of errors and biases, especially those arising from differences in the experimental and study designs (10). These problems are reflected in equivocal or inconsistent results and may lead researchers to design inappropriate follow-up studies or medical applications. Accordingly, we have collected association studies comprehensively, classified the studies by ethnicity of subjects, re-evaluated the associations by unified significance level and assessed the associations by population-based reproducibility. All data are publicly available in a regularly updated web database, RAvariome. Additionally, an online tool for predicting the genetic risk of RA for an individual was developed to support further analysis for preventive intervention of genetic RA risk carrier.

## Collection and Extraction of Data

**Figure 1.**
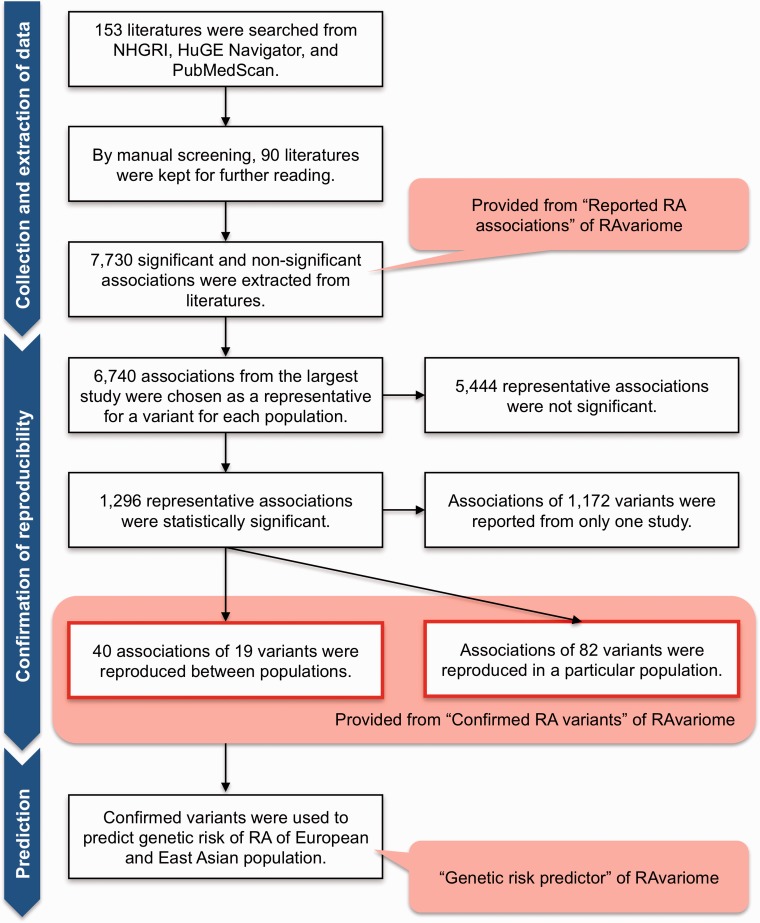
The flowchart for the assessment of association results from articles. From comprehensive search by NHGRI Genome-Wide Association catalog, HuGE Navigator and PubMedScan, 153 literatures were collected. In all, 7730 associations were collected from 90 literatures by manual screening and reading. The associations were grouped into the nine geographical populations based on sample information. A total of 6740 associations (6614 unique genetic variants) were chosen as representative associations of the variants in either population. In all, 5444 representative associations (5405 unique variants) did not reach significance level, and 1296 representative associations (1274 unique variants) were statistically significant and remained for further analysis. Forty representative associations of 19 variants were reproduced between different populations, and associations of 82 variants were reproduced in the particular population. The confirmed variants were used to predict genetic risk of RA based on individual’s genotype. The data can be view and downloaded from the web database RAvariome.

We collected 153 English-language literatures from the NHGRI catalog and HuGE Navigator and the automatic paper recommendation system PubMedScan (http://medals.jp/pubmedscan/) (Figure 1). Then, manual screening of their abstracts excluded articles about other autoimmune diseases, pharmacogenomics and gene environment studies. After filtering, 90 literatures about GWASs, fine mapping studies and meta-analyses were kept for further reading. Not only statistically significant but also statistically not significant association results, ethnicity of subjects, the country where subjects were recruited, the total number of cases and controls, the analysis platform and the study design were extracted manually from full text, tables or supplementary data. The association results of human genetic variants included SNPs, HLA alleles, copy number variations and variable number of tandem repeats markers. Finally, 7730 association results were stored in a database.

The results of statistical tests in original literatures were re-evaluated according to the following criteria. We determined two kinds of significance levels for GWAS that assayed genome-wide SNPs and for follow-up study, such as meta-analysis, that assayed only few SNPs of interest. For a GWAS, if the corresponding *P*-value was <5.0 × 10^−8^, the result of the statistical analysis was judged as significant evidence of strong association, and *P-*value between 1.0 × 10^−5^ and 5.0 × 10^–8^ was judged as significant evidence of moderate association. Associations with *P* > 1.0 × 10^−^^5^ were judged as not significant. For follow-up study, associations were judged as ‘strong’ for *P* < 0.01, ‘moderate’ for *P*-value between 0.01 and 0.05 and ‘not significant’ for *P* > 0.05. Exceptionally, a meta-analysis of GWAS result and combined analysis of GWAS and replication results were judged by genome-wide significance threshold. According to our significance level, 5970 associations were classified as statistically not significant, and 1760 associations were classified as either strong or moderate associations.

## Confirmation of Reproducibility of RA Risk Variants

To confirm reproducibility of genetic associations based on a geographical information, all associations including non-significant results were grouped by the following nine populations; European (including European American, European Australian, European New Zealander, West European, North European, South European and East European), East Asian (including South-East Asian), West Asian, South Asian, South American, Central American, North African, South African and African American. Because many follow-up studies of previous GWAS researches were conducted, the representative association for the variant in each geographical population was selected from the result of a study of the largest number of case subjects. Out of 6740 representative associations, 1296 were statistically significant and remained for further reproducibility assessment. Notably, 34 variants reached significant level in small-scale studies, but were not confirmed by larger studies.

The genetic associations were classified into two classes based on reproducibility between or within population. To assess reproducibility of an association between different populations, the variants that was reproduced by independent representative associations were identified. A genetic variant that showed opposite direction of association between different populations was excluded. Accordingly, 40 representative associations of 19 variants located in 12 loci were confirmed as ‘inter-population reproduced’ based on independent studies of different populations such as African American, Central American, East Asian, European, South American, South Asian and West Asian (Table 1).

**Table 1.** List of RA risk variants confirmed between different populations

Related genes	SNP-allele	*P*-value	Genotyping coverage	OR	Case/control numbers	Population	References
*APOM*	rs805297-A	6.65e-005	Selected	1.40	578/711	East Asian	Hu HJ, Exp Mol Med, 2011 (11)
	rs805297-A	1.49e-016	GW		1147/1079	European	Padyukov L, Ann Rheum Dis, 2011 (12)
*CCR6*	rs3093023-A	2.1e-017	GW	1.27	4074/16 891	East Asian	Okada Y, Nat Genet, 2012 (13)
	rs3093023-A	1.5e-011	GW	1.12	12 307/28 975	European	Stahl EA, Nat. Genet, 2010 (14)
*CTLA4, ICOS*	rs231775-G	0.01	Selected	1.45	199/199	Central American	Li X, J Clin Immunol, 2012 (15)
	rs231775-G	0.002	Selected (Meta)	1.18	2734/1441	East Asian	Li X, J Clin Immunol, 2012 (15)
	rs231775	0.009	Selected (Meta)	1.07	6160/6684	European	Plant D, Ann Rheum Dis, 2010 (16)
*HLA-DRB1*	rs13192471-G	1.9e-058	GW	1.97	2303/3380	East Asian	Kochi Y, Nat. Genet, 2010 (17)
	rs13192471-G	6.7e-016	Selected	2.16	983/1007	South Asian	Prasad P, PLoS One, 2012 (18)
	rs2157337-C	2.6e-118	GW	1.99	4074/16 891	East Asian	Okada Y, Nat Genet, 2012 (13)
	rs2157337-C	1e-299	GW	2.50	5539/20 169	European	Stahl EA, Nat Genet, 2010 (14)
	rs2516049-C	3.6e-031	GW	2.18	874/855	East Asian	Terao C, PLoS One, 2011 (19)
	rs2516049-C	1.64e-042	GW		1147/1079	European	Padyukov L, Ann Rheum Dis, 2011 (12)
	rs6457617-T	3.44e-076	GW		1861/2938	European	WTCCC, Nature, 2007 (20)
	rs6457617-A	1.6e-009	Selected	1.48	983/1007	South Asian	Prasad P, PLoS One, 2012 (18)
	rs660895-G	5.83e-053	GW		1147/1079	European	Padyukov L, Ann Rheum Dis, 2011 (12)
	rs660895-G	2.56e-005	Selected	1.52	983/1007	South Asian	Prasad P, PLoS One, 2012 (18)
	rs6910071-G	1e-299	GW	2.88	5539/20 169	European	Stahl EA, Nat Genet, 2010 (14)
	rs6910071	0.04	Selected	1.27	983/1007	South Asian	Prasad P, PLoS One, 2012 (18)
*IKZF3**^a^*	rs2872507-G	0.006	Selected	0.79	440/795	African American	Kurreeman FA, Am J Hum Genet, 2012 (21)
	rs2872507-G	0.003	Selected	0.91	2414/14 245	East Asian	Kurreeman FA, Am J Hum Genet, 2012 (21)
	rs2872507-A	9.4e-007	GW	1.09	12 307/28 975	European	Stahl EA, Nat Genet, 2010 (14)
*IL2, IL21*	rs13119723-G	6.8e-007	GW	0.88	12 307/28 975	European	Stahl EA, Nat Genet, 2010 (14)
	rs13119723-G	0.008	Selected	0.75	983/1007	South Asian	Prasad P, PLoS One, 2012 (18)
	rs6822844	5.89e-008	Selected (Meta)	0.88	12953/13370	European	Plant D, Ann Rheum Dis, 2010 (16)
	rs6822844	0.019	Selected	0.61	354/368	South American	Maiti AK, Arthritis Rheum, 2010 (22)
*IL2RA*	rs2104286	2.48e-05	Selected (Meta)	0.91	10 112/10 450	European	Plant D, Ann Rheum Dis, 2010 (16)
	rs2104286-G	0.00019	Selected	0.73	983/1007	South Asian	Prasad P, PLoS One, 2012 (18)
*IL6*	rs1800795-G	1e-008	Selected	0.074	120/168	East Asian	Lee YH, Inflamm Res, 2012 (23)
	rs1800795-G	7.4e-005	Selected	0.46	425/247	West Asian	Lee YH, Inflamm Res, 2012 (23)
*OLIG3, TNFAIP3*	rs2230926	0.032	Selected	1.847	148/1513	African American	Lee YH, Inflamm Res, 2012 (23)
	rs2230926-C	1.6e-006	GW	1.31	7069/15 876	East Asian	Kochi Y, Nat Gen*et, 2010 (17)
	rs5029937-T	3.9e-009	GW	1.33	4074/16 891	East Asian	Okada Y, Nat Genet, 2012 (13)
	rs5029937	4.62e-010	Selected (Meta)	1.42	7731/9403	European	Plant D, Ann Rheum Dis, 2010 (16)
*SPRED2*	rs934734-G	3.2e-007	GW	1.19	4074/16 891	East Asian	Okada Y, Nat Genet, 2012 (13)
	rs934734-G	5.3e-010	GW	1.13	12 307/28 975	European	Stahl EA, Nat Genet, 2010 (14)
*STAT4*	rs7574865-T	1.8e-006	GW	1.17	7069/15 876	East Asian	Kochi Y, Nat Genet, 2010 (17)
	rs7574865	2.99e-015	Selected (Meta)	1.18	14 394/16 131	European	Plant D, Ann Rheum Dis, 2010 (16)
*ZEB1**^a^*	rs2793108-T	0.001	Selected	1.11	2414/14 245	East Asian	Kurreeman FA, Am J Hum Genet, 2012 (21)
	rs2793108-C	0.006	Selected	0.84	983/1007	South Asian	Prasad P, PLoS One, 2012 (18)

^a^Confirmed RA risk loci that were not mentioned in RA reviews (10, 24–27). Related genes, refSNP ID and allele, association *P*-value, study type to set significance level, OR, sample sizes of case/control, geographical population of samples and references are shown. Genotyping coverage indicated whether the study assayed genome-wide (GW) SNPs or selected SNPs.

To assess reproducibility of an association within the particular population, we identified the statistically significant representative association that was reproduced by an independent study of the same population. As a result, associations of 82 variants were confirmed as ‘intra-population reproduced’. Intra-population reproduced variants were only reported in European and East Asian populations (53 SNPs and 29 SNPs, respectively).

## Prediction of Genetic RA Risk

To facilitate personalized medicine and preventive health care, a tool to calculate genetic risk of RA for an individual was developed based on confirmed RA risk variants (intra- and inter-population-reproduced variants). To avoid overestimation, if confirmed RA risk variants are closely located to each other, i.e. in the same linkage disequilibrium block, only the genetic variant with a smaller *P*-value was used as a risk marker of that locus.

The genetic risk score (GRS) and the relative genetic risk (RGR) of RA for an individual were calculated by the combination of RA risk markers. As described in previous studies, an unweighted GRS, simply counting the number of risk alleles carried by an individual, was not applicable to RA because HLA-DRB1 alleles had substantially higher odds ratio (OR) than alleles at non-HLA loci (28–30). Accordingly, we used a weighted GRS that increases additively by log-OR of the risk allele (31). GRS was calculated as follows:

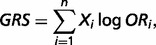

where *n* is the number of markers available and *X_i_* represents the copy number of risk allele at a marker *i*. If an individual carries 1 risk allele at *i*th marker, *X_i_* is 1, and if an individual carries 2 risk alleles, then *X_i_* is 2.

To calculate RGR of the average population for each marker, an individual genotype-specific risk *s_i_* is estimated as:

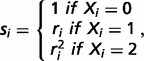

where *r_i_* is a risk factor assumed to equal the allelic OR of *i*th marker. RGR of individual marker is estimated as:

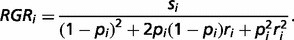



Here *p_i_* is the risk allele frequency of *i*th marker in the control group. Thus, the overall RGR of an individual estimated by multi-markers is calculated as:

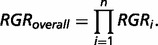



## RAvariome Web Interface and Usage

To provide our results and a genetic risk prediction tool for researchers and clinicians, a web database RAvariome was developed. By comparing number of literatures and association data with other existing literature-based GWAS databases, RAvariome provides the most comprehensive dataset of associations of RA (Table 2). RAvariome consists of four sections: overview of RA, the list of confirmed RA variants, the collection of reported RA associations and genetic risk prediction tool. In the overview section, the heritability, environmental factors and protective factors of RA are described.

**Table 2.** Comparing the number of literatures and associations of RAvariome with other literature-based databases

Database	Condition of literature collection	Number of literatures	Condition of data collection	Number of associations
RAvariome	RA GWAS and follow-up studies	90	All associations described in literature	7730
NHGRI GWAS catalog (5)	GWAS literature	14^a^	Associations reached *P*-values <1.0 × 10^−5^	96^a^
HuGE Navigator (8)	GWAS literature	40^a^		84^a^
	meta-analysis	98^a^		
GWASdb (6)	GWAS literature and dataset from GAD		Associations reached *P-*values <1.0 × 10^−3^	1748^b^

^a^The number for HuGE Navigator and NHGRI GWAS catalog were obtained by searching the term ‘rheumatoid arthritis’ at 12 March 2013. ^b^For GWASdb, number of data was the result as of July 2012.

**Figure 2.**
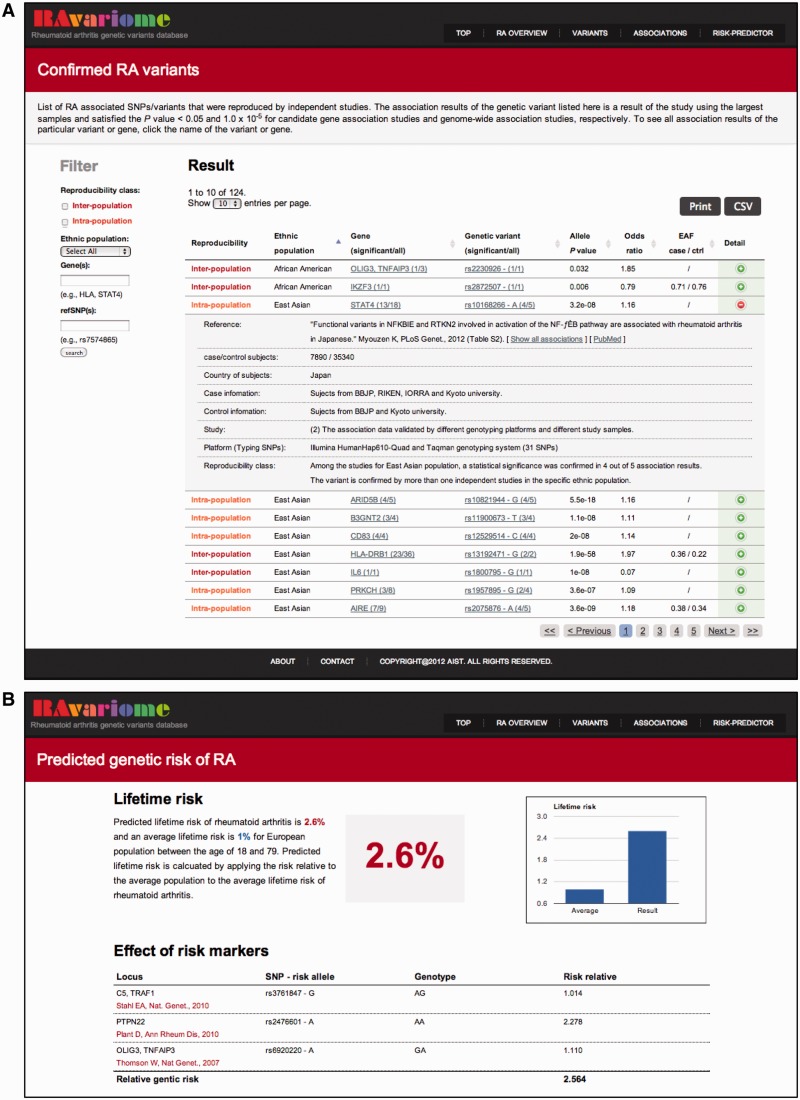
Screenshots of web database RAvariome. (**A**) View of the confirmed RA variants of RAvariome. In the left sidebar, users can search an association data by reproducibility class of genetic variants, population of subjects, genes and refSNP ID. The query results will be shown in a tabular format. Users can get detailed study information by click on the detail icon on rightmost column. The results can be easily downloaded as print format and comma-separated values (CSV) format. (**B**) The result of the genetic RA risk predictor. Lifetime risk and effects of each risk markers based on selected genotypes are shown.

The page entitled ‘Confirmed RA variants’ provides users a list of genetic variants with the reproducibility class (Figure 2A). The list provides detailed study information of the representative association of the specific variant and the particular geographical population. In this section, users can search the confirmed RA risk variants by the reproducibility class, the geographical population of the subjects, a gene name or refSNP ID.

The page entitled ‘Reported RA associations’ leads users to a list of all statistically significant and statistically not significant associations described in literatures. Users can search association data by study design, geographical population of the subjects, nationality of the subjects, number of subjects and sub-phenotype of cases such as cases with 100% seropositive for rheumatoid factor and/or anti-CCP.

The page entitled ‘Genetic risk predictor’ provides a tool for predicting the genetic risk of RA calculated by the ethnicity and genotypes of an individual (Figure 2B). By choosing a population from either European or East Asian, the confirmed RA risk markers and their common three genotypes are displayed in the list. Users can select one genotype for each risk marker, and selected genotypes will be used for estimation of RGR and GRS. RGR is the index that assumed the risk of average population as baseline. GRS is the index that assumed non-risk allele carrier as baseline and used in basic researches. For example, if genotypes AG for rs3761847, AA for rs2476601 and GA for rs6920220 are selected at the European population, a predicted lifetime risk will be 2.6%, 2.6-fold higher than the prevalence of RA in general European population (1%).

## Discussion

RAvariome was developed to provide a list of RA-associated genetic variants with the degree of reproducibility, based on comprehensive re-evaluated results of the genetic association studies. By comparing our results with reviews (10, 24–27) of RA, we found that two loci from inter-population-reproduced variants were not reported in reviews and have been validated in recent meta-analyses (Table 1). Thus, we conclude that our database provides the newest and most reliable genetic risk variants based on the comparative verification.

As the intra-population-reproduced variants were observed only in European and East Asian populations, we used gene set enrichment analysis (32) to discover common functional mechanisms underlying different gene sets confirmed specifically in European and East Asian populations. From inter- and intra-reproduced-variants, 10 genes were confirmed in both European and East Asian populations, although 39 and 30 genes were confirmed only in the European and East Asian populations, respectively. Interestingly, the European and East Asian unique gene sets had the following gene sets in common: genes involved in immune system, gene upregulated by CD40 signaling in Ramos cells, genes upregulated in TK6, WTK1 and NH32 cell lines in response to ionizing radiation, genes modulated in HeLa cells by TNF via NFKB pathway, genes whose promoters are bounded by FOXP3 based on a chromatin immunoprecipitation (ChIP)-chip analysis, genes involved in cytokine signaling in immune system and genes downregulated in freshly isolated CD31− versus the CD31+ counterparts (Table 3). This result suggested that even if the same genes were not reproduced between European and East Asian populations, several pathways were common in both populations.

**Table 3.** Result of gene set enrichment analysis using population unique RA risk genes that was confirmed only in European and East Asian populations, respectively

Description of MSigDB gene set (number of genes in gene set)	European unique 39 genes			East Asian unique 30 genes		
	Genes in overlap	*P*-value	FDR q-value^a^	Genes in overlap	*P*-value	FDR q-value^a^
Genes involved in Immune System (933)	*CD40, HLA-DPB1, IRF5, CD28, IL2, IL2RA, CD226, IL2RB, TNFRSF14, IL6ST, C5, PRKCQ, REL, KIF5A*	3.89E-15	2.34E-11	*NFKBIE, IL6, IRF8, IRF4, CSF2, PTPN2*	8.13E-06	2.26E-03
Genes upregulated by CD40 (GeneID = 958) signaling in Ramos cells (EBV negative Burkitt lymphoma) (101).	*CD40, HLA-DPB1, IRF5, CD58, BATF, TRAF1, SH2B3*	1.45E-12	4.35E-09	*CD83, NFKBIE, ARID5B, IRF4*	2.54E-07	2.18E-04
Genes upregulated in TK6, WTK1 and NH32 cell lines (lymphoblast) in response to ionizing radiation (149).	*IL2RB, BATF, TRAF1, CCL21, CDK6, PTPN22*	1.74E-09	8.73E-07	*CD83, NFKBIE, IRF4*	7.01E-05	1.11E-02
Genes involved in cytokine signaling in immune system (270).	*HLA-DPB1, IRF5, IL2, IL2RA, IL2RB, IL6ST*	6.04E-08	1.91E-05	*IL6, IRF8, IRF4, CSF2, PTPN2*	3.05E-07	2.29E-04
Genes whose promoters are bound by FOXP3 (GeneID = 50943) based a ChIP-chip analysis (491).	*CD28, IL2RA, CD226, IL6ST, PRKCQ, PTPN22, ANKRD55*	8.89E-08	2.67E-05	*ETS1, ARID5B, B3GNT2, IRF4, PRKCH, FLI1, EXOC2*	5.66E-09	1.13E-05
Genes upregulated in the normal-like subtype of breast cancer (476).	*CD40, PRKCQ, HLA-DRA, CD2, CCL21, PTPN22*	1.66E-06	2.68E-04	*IL6, IRF8, FLI1, PDE2A*	1.15E-04	1.47E-02
Genes upregulated in prostate cancer samples from African-American patients compared with those from the European-American patients.	*HLA-DPB1, CD28, HLA-DRA, CD2, PTPN22*	3.38E-06	4.83E-04	*CD83, IRF8, FLI1*	5.43E-04	3.82E-02
Genes from common genomic gains observed in a meta-analysis of copy number alterations across a panel of different cancer cell lines and tumor samples (323).	*IL2, REL, CDK6, DDX6, AFF3*	4.91E-06	6.29E-04	*IRF4, FLI1, MN1*	6.79E-04	4.39E-02
Calcineurin-regulated NFAT-dependent transcription in lymphocytes (47).	*IL2, IL2RA, PRKCQ*	6.72E-06	8.24E-04	*IRF4, CSF2*	2.95E-04	2.70E-02
Genes downregulated in freshly isolated CD31− [GeneID = 5175] (stromal stem cells from adipose tissue) versus the CD31+ (non-stem) counterparts (216).	*HLA-DPB1, SH2B3, HLA-DRA, PODXL*	2.36E-05	2.49E-03	*PRKCH, FLI1, ANXA3, PDE2A*	5.27E-06	1.90E-03
Genes modulated in HeLa cells (cervical carcinoma) by TNF [GeneID = 7124] via NFKB pathway (28).	*REL, TRAF1*	2.17E-04	1.55E-02	*CD83, NFKBIE, GCH1, IL6*	1.31E-09	7.87E-06
Cytokines and inflammatory response (29).	*IL2, HLA-DRA*	2.33E-04	1.57E-02	*IL6, CSF2*	1.11E-04	1.47E-02

^a^The gene set enrichment analysis was performed by computing overlaps between the population unique genes and gene sets of positional (c1), curated (c2) and motif (c3) of MSigDB (32). The MSigDB gene sets listed in left column were significantly enriched in both populations at 5% false discovery rate (FDR) q-value.

The purpose of open access database RAvariome is to be a standard resource not only for RA researchers but also for RA clinicians and the general public. RAvariome is therefore designed as simple as possible to get confirmed RA genetic risk variants for each geographical population. With ongoing progress in sequencing technology, the number of genetic studies of RA will continue to grow. RAvariome will be periodically updated in concert with progress in RA genetic research and will incorporate a new genetic risk prediction method (33). An open access resource would be valuable to raise the precision of the clinical genetic tests and to develop effective prevention programs of RA based on genetic and population differences among individuals.
